# Ubiquitin E3 ligase SPOP is a host negative regulator of enterovirus 71-encoded 2A protease

**DOI:** 10.1128/jvi.00786-23

**Published:** 2023-10-05

**Authors:** Lichao Zang, Xinyu Yang, Yan Chen, Fan Huang, Yukang Yuan, Xiangjie Chen, Yibo Zuo, Ying Miao, Jin Gu, Hui Guo, Wenxin Xia, Yang Peng, Mengyuan Tang, Ziwei Huang, Yangyang Wang, Jinhong Ma, Jingting Jiang, Wei Zhou, Hui Zheng, Weifeng Shi

**Affiliations:** 1 Department of Laboratory Medicine, The Third Affiliated Hospital of Soochow University, Changzhou, Jiangsu, China; 2 Department of Clinical Laboratory, The First Affiliated Hospital of Ningbo University, Ningbo First Hospital, Ningbo, Zhejiang, China; 3 International Institute of Infection and Immunity, Institutes of Biology and Medical Sciences, Soochow University, Suzhou, Jiangsu, China; 4 Jiangsu Key Laboratory of Infection and Immunity, Soochow University, Suzhou, Jiangsu, China; 5 Department of Tumor Biological Treatment, The Third Affiliated Hospital of Soochow University, Changzhou, Jiangsu, China; University of Kentucky College of Medicine, Lexington, Kentucky, USA

**Keywords:** SPOP, EV71, 2A protease, ubiquitination

## Abstract

The 2A protease (2A^pro^) encoded by enterovirus 71 (EV71) serves as an accessory protein with significant involvement in the regulation of EV71 infection and viral replication. EV71-2A^pro^ exhibits the ability to suppress various host factors, thereby disrupting the cellular antiviral immune response. However, whether host factors downregulate EV71-2A^pro^ remains largely unknown. Here, we discovered that the speckle-type POZ protein (SPOP), functioning as a host E3 ubiquitin ligase, triggers ubiquitination modifications and subsequent degradation of EV71-2A^pro^. SPOP interacts with EV71-2A^pro^ and exhibits a dose-dependent downregulation of EV71-2A^pro^ levels. Conversely, knockdown of endogenous SPOP upregulated EV71-2A^pro^ levels. Subsequent investigations revealed that the SPOP facilitates the lysosome-dependent degradation of EV71-2A^pro^ by inducing K48-linked polyubiquitination of EV71-2A^pro^, which ultimately restricts EV71 replication. These findings shed light on the ubiquitination and lysosome-dependent regulation of the crucial EV71-encoded protease, offering a potential therapeutic target for the treatment of EV71 infection.

**IMPORTANCE:**

EV71 poses a significant health threat to children aged 5 and below. The process of EV71 infection and replication is predominantly influenced by ubiquitination modifications. Our previous findings indicate that EV71 prompts the activation of host deubiquitinating enzymes, thereby impeding the host interferon signaling pathway as a means of evading the immune response. Nevertheless, the precise mechanisms by which the host employs ubiquitination modifications to hinder EV71 infection remain unclear. The present study demonstrated that the nonstructural protein 2A^pro^, which is encoded by EV71, exhibits ubiquitination and degradation mediated by the host E3 ubiquitin ligase SPOP. In addition, it is the first report, to our knowledge, that SPOP is involved in the host antiviral response.

## INTRODUCTION

Enterovirus 71 (EV71) was initially isolated and characterized in 1969. It is classified as a member of the enterovirus genus within the *Picornavirus* family, possessing a positive single-stranded genome of approximately 7.4 kb in length. Currently, EV71 has become one of the main fatal viruses for those under 5 years old, with some severe complications in hand, foot, and mouth disease (HFMD), such as acute flaccid paralysis, brainstem encephalitis, neurological pulmonary edema, and even death (1, 2). During the course of virus evolution, EV71 has employed numerous strategies to evade immune attack, thereby promoting its survival and replication (3 – 5). EV71-encoded proteins not only extensively exploit signal transduction processes in host cells but also impair antiviral immune responses (6 – 12). Nonetheless, host cells have developed diverse mechanisms to counteract the detrimental effects of EV71 on antiviral activities (13 – 15).

Initially, EV71 encodes a polyprotein precursor, which is subsequently cleaved by the nonstructural proteins 2A protease (2A^pro^) and 3C protease (3C^pro^). This process leads to the production of mature viral proteins, comprising four structural proteins (VP1, VP2, VP3, and VP4) and seven nonstructural proteins (2A, 2B, 2C, 3A, 3B, 3C, and 3D) (16 – 18). These nonstructural proteins play a significant role in various stages of viral replication. Specifically, EV71-2A^pro^, which has a molecular weight of 15 kDa, is known to be crucial in viral replication. Structural analysis revealed that 2A^pro^ possesses cysteine protease activity, enabling it to cleave at its own N-terminus at the junction between VP1 and 2A of the polyprotein (19). The protease active site of EV71-2A^pro^ consists of the catalytic triads C110A, H21, and D39 (20). Interestingly, previous studies have demonstrated that EV71-2A^pro^ is capable of manipulating host cell gene expression through the cleavage of various proteins, such as eIF4GI, eIF4GII, PABP, and P-body (6, 21, 22). In response to antiviral cellular immune defense, host cells accumulate a significant number of proteins and RNAs in the cytoplasm, resulting in the formation of envelope-free emergency granules known as stress granules (SGs) (23). The interaction between eIF4GI-RasGAP SH3 domain binding protein plays a crucial role in the conversion of typical stress granules (tSGs) into atypical stress granules (aSGs), thereby facilitating the infection caused by EV71 (21, 24). Moreover, EV71-2A^pro^ has the capability to impede the production of type I interferon (IFN) and the subsequent IFN signaling pathway, thereby evading the host’s innate immune response. This is achieved through the cleavage of melanoma differentiation-associated protein 5 (MDA5) and mitochondrial antiviral-signaling protein (MAVS), which effectively inhibits IFN production (25). Additionally, EV71-2A^pro^ disrupts the IFN signaling pathway by targeting indirect interaction with IFN receptor subunit 1 (IFNAR1) (26). Furthermore, the induction of cell death by EV71-2A^pro^ can occur through the inhibition of host protein translation or the upregulation of thioredoxin-interacting protein, leading to cellular apoptosis (27). Despite these discoveries shedding light on the mechanisms of EV71-2A^pro^ in the downregulation of host proteins, the manner in which host factors downregulate EV71-2A^pro^ remains largely unexplored.

The speckle-type POZ (poxvirus and zinc finger protein) protein (SPOP) belongs to the BTB/POZ protein family and was initially discovered as a dispersed protein resembling dots within the nucleus in the sera of scleroderma patients in 1997. It exhibits extensive expression in diverse tissues and organs (28). The SPOP protein is composed of 374 amino acid residues, which encompass the N-terminal MATH domain, BTB/POZ domain, BACK domain, and C-terminal nuclear localization sequence (NLS). The MATH binding domain, containing specific amino acids, plays a crucial role in the specific recognition and binding of substrate proteins (29). The Cullin ring ligase family, the largest E3 ubiquitin ligase family, consists of Cullin family proteins, active ring domain proteins Rbx1 or Rbx2, and aptamer proteins (30, 31). The combination of SPOP with cullin3-Rbx1 forms an E3 ubiquitin ligase complex, which is involved in various cellular biological processes, including the immune response (32), tumorigenesis (33), development (34), and transcriptional regulation (35). Previous studies have demonstrated that SPOP acts as a tumor suppressor in prostate (36) and endometrial cancers (37), while it exhibits a tumor-promoting role in kidney cancer (38). In addition, SPOP, functioning as a ubiquitin E3 ligase, facilitates the process of K48-linked polyubiquitination and subsequent proteasome-mediated degradation of myeloid differentiation primary response protein 88 (MyD88). This exerts a negative regulatory effect on NF-κB activation and the production of proinflammatory cytokines (39 – 41). Nevertheless, the extent to which SPOP regulates viral infection and the underlying mechanisms involved remain undisclosed.

In the present study, our findings demonstrated that SPOP exerts a negative effect on EV71-2A^pro^ protein stability, consequently leading to a reduction in EV71-2A^pro^ protein levels. Moreover, we elucidated the underlying mechanism through which SPOP facilitates the degradation of EV71-2A^pro^ and inhibition of EV71 infection. Thus, this study offers prospective therapeutic approaches for managing EV71 infection in clinical settings.

## RESULTS

### EV71-2A^pro^ interacts with the host ubiquitin E3 ligase SPOP

**Fig 1:**
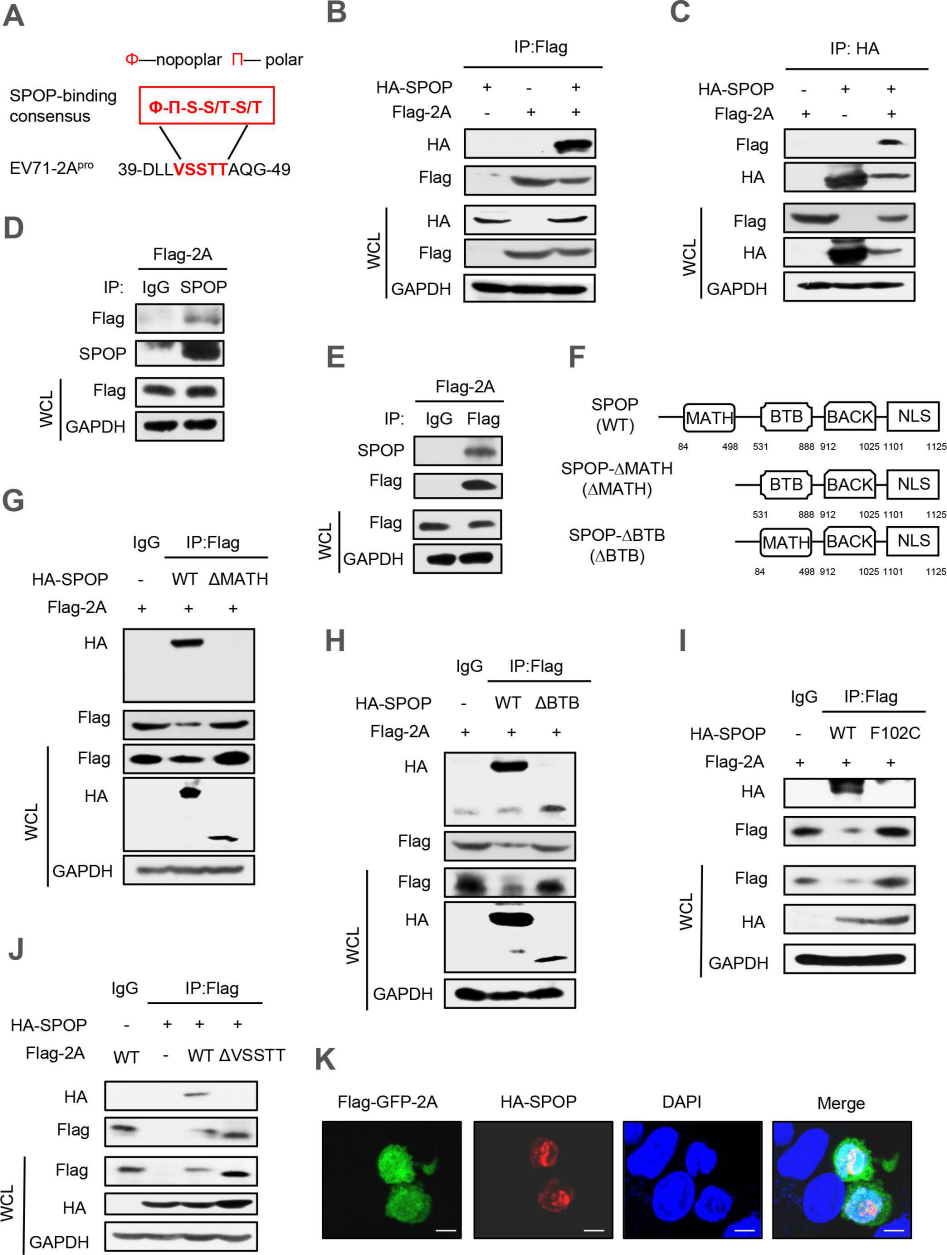
EV71-2A^pro^ interacts with the host ubiquitin E3 ligase SPOP. (**A**) Alignment of a putative SPOP-binding motif in EV71-2A^pro^. (**B and C**) Immunoprecipitation analysis of the interaction between SPOP and 2A^pro^ in HEK293T cells co-transfected with HA-SPOP and Flag-2A plasmids. WCL, whole cell lysate. (**D**) HEK293T cells were transfected with a vector to express Flag-2A. Immunoprecipitation was performed using the Flag affinity gel, and then endogenous SPOP was detected by immunoblotting with an anti-SPOP antibody. (**E**) HEK293T cells were transfected with a vector to express Flag-2A. Immunoprecipitation was performed using the SPOP antibody, and Flag-2A was detected by immunoblotting with an anti-Flag antibody. (**F**) The domains of the SPOP protein and SPOP deletion mutants. (**G, H, and I**) Immunoprecipitation analysis of the interaction between SPOP mutants and 2A^pro^ in HEK293T cells co-transfected with Flag-2A and either HA-SPOP wild type (WT), HA-SPOP-ΔMATH mutant, HA-SPOP-ΔBTB mutant or HA-SPOP-F102C mutant plasmids. (**J**) Immunoprecipitation analysis of the interaction between SPOP and 2A^pro^ in HEK293T cells co-transfected with HA-SPOP and Flag-2A (WT or the ΔVSSTT mutant). (**K**) Immunofluorescence analysis of the co-localization of HA-SPOP and Flag-GFP-2A in HeLa cells. Data are representative of three independent experiments with similar results (**B–J**).

To examine the degradation of EV71-2A^pro^ within host cells, an initial analysis was conducted on the amino acid sequences of EV71-2A^pro^. We found that EV71-2A^pro^ possesses the amino acid sequence VSSTT. This site aligns with the consensus motif (Ø-Π-S-S/T-S/T) targeted by the E3 ubiquitin ligase SPOP (Fig. 1A). Interestingly, we noticed that this VSSTT sequence is fairly conserved in many different strains of the EV71-A subtype and even many types of enteroviruses. Therefore, an immunoprecipitation analysis was conducted to investigate the potential interaction between EV71-2A^pro^ and SPOP. The results showed that the SPOP is able to interact with the 2A^pro^ of EV71, as evidenced by the observed interaction between HA-SPOP and Flag-2A in cells (Fig. 1B and C). Additionally, we provided evidence that endogenous SPOP is also capable of interacting with 2A^pro^ (Fig. 1D and E).

The SPOP protein comprises 374 amino acids and encompasses 4 distinct structural domains, namely, the N-terminal MATH structural domain, the BTB/POZ structural domain, the BACK structural domain, and the C-terminal NLS structural domain (29). The MATH structural domain assumes a crucial function in substrate recognition and recruitment, whereas the BTB/POZ structural domain plays a pivotal role in the operation of E3 ubiquitin ligase (42, 43). Hence, we generated deletion mutants of SPOP, including HA-SPOP-ΔMATH (SPOP-Δ84–498) and HA-SPOP-ΔBTB (SPOP-Δ531–888), by deleting the functional structural domains of SPOP (Fig. 1F). Our results showed that the absence of the MATH domain in SPOP prevents its binding to Flag-2A, while the deletion of the BTB/POZ domain does not hinder its interaction with Flag-2A (Fig. 1G and H).

All somatic mutations of SPOP identified in prostate cancers, including Y87C, F102C, W131G, and F133V, are situated within the MATH domain and demonstrate a dominant-negative influence on substrate binding and degradation (44, 45). Accordingly, we produced the SPOP-F102C mutant to verify the consequences of this mutation on the interaction between SPOP and EV71-2A^pro^. In accordance with prior studies, the F102C mutant of SPOP exhibited an inability to interact with Flag-2A (Fig. 1I). In addition, we deleted the VSSTT amino acids to obtain the EV71-2A-ΔVSSTT mutant. We found that there was no interaction between SPOP and EV71-2A-ΔVSSTT (Fig. 1J). Furthermore, immunofluorescence microscopy revealed noticeable co-localization of HA-SPOP and Flag-GFP-2A in cells (Fig. 1K). Collectively, these findings demonstrated the interaction between EV71-2A^pro^ and SPOP.

### SPOP is a cellular negative regulator of EV71-2A^pro^

**Fig 2:**
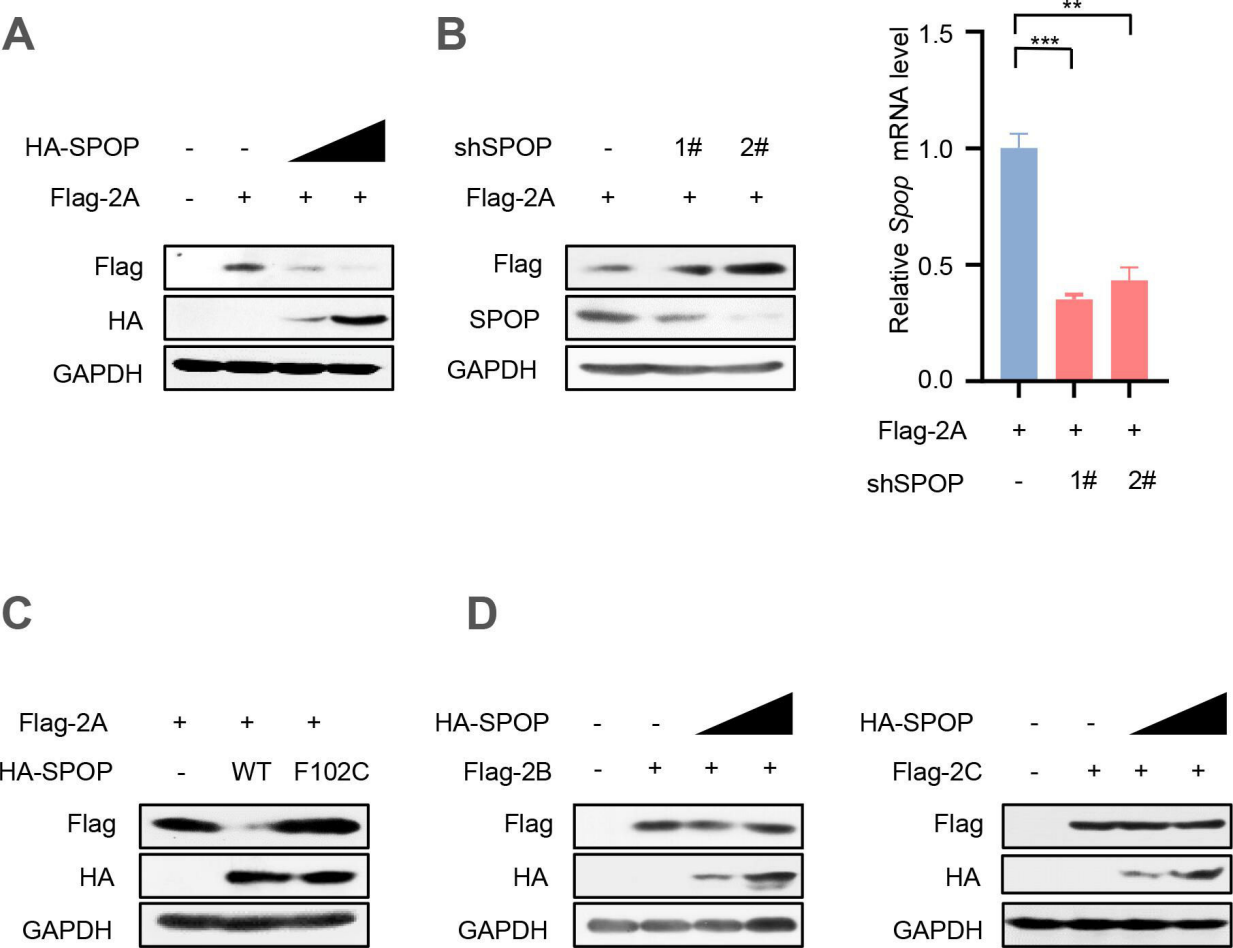
SPOP is a negative regulator of EV71-2A^pro^. (**A**) HEK293T cells were co-transfected with vectors to express Flag-2A and increased amounts (0.5 and 1.0 µg) of HA-SPOP. Whole cell extracts were subjected to immunoblotting as indicated. (**B**) HEK293T cells were co-transfected with vectors to express Flag-2A and control shRNAs (−) or two shRNAs against SPOP (shSPOP, 1# and 2#). Seventy-two hours after transfection, whole cell extracts were analyzed by immunoblotting using the indicated antibodies (left). The relative mRNA levels of SPOP were measured by RT-qPCR in HEK293T cells transfected with control shRNA (−) or shSPOP (1# and 2#) (right). (**C**) HEK293T cells were transfected with vectors to express Flag-2A and either HA-SPOP wild type (WT) or HA-SPOP-F102C mutant plasmids. Flag-2A protein levels were analyzed by immunoblotting as indicated. (**D**) HEK293T cells were transfected with either Flag-2B or Flag-2C plasmids, together with empty vectors (−) or increasing amounts of HA-SPOP plasmids. Whole cell extracts were analyzed by immunoblotting using the indicated antibodies. Data are representative of three independent experiments with similar results (**A–D**).

In light of the interaction between SPOP and 2A^pro^, we questioned whether SPOP could regulate the levels of EV71-2A^pro^. Thus, HEK293T cells were transfected with two vectors expressing Flag-2A or HA-SPOP. The results demonstrated that overexpression of SPOP significantly reduced the levels of Flag-2A protein in a dose-dependent manner (Fig. 2A). Subsequently, two specific short hairpin RNAs (shRNAs) targeting human SPOP (shSPOP) were employed to reduce endogenous SPOP. The results showed a significant upregulation of Flag-2A protein levels upon knockdown of SPOP (Fig. 2B). Additionally, the impact of the SPOP-F102C mutant on Flag-2A protein levels was also investigated. Consistent with previous findings, SPOP carrying the F102C mutation failed to reduce the protein levels of Flag-2A in comparison to SPOP-WT (Fig. 2C).

The P2 precursor protein encoded by EV71 gives rise to three nonstructural proteins, namely, EV71-2A^pro^, 2B, and 2C (17). Thus, we investigated whether the protein levels of these EV71-encoded proteins could also be influenced by SPOP. To address this, we transfected HEK293T cells with Flag-2B or Flag-2C plasmids, along with HA-SPOP plasmids. Our results showed that SPOP does not exert a significant impact on the levels of these EV71-encoded proteins (Fig. 2D). In conclusion, these findings provide evidence that cellular SPOP functions as a negative regulator of EV71-2A^pro^ protein levels.

### SPOP lowers EV71-2A^pro^ protein stability

**Fig 3:**
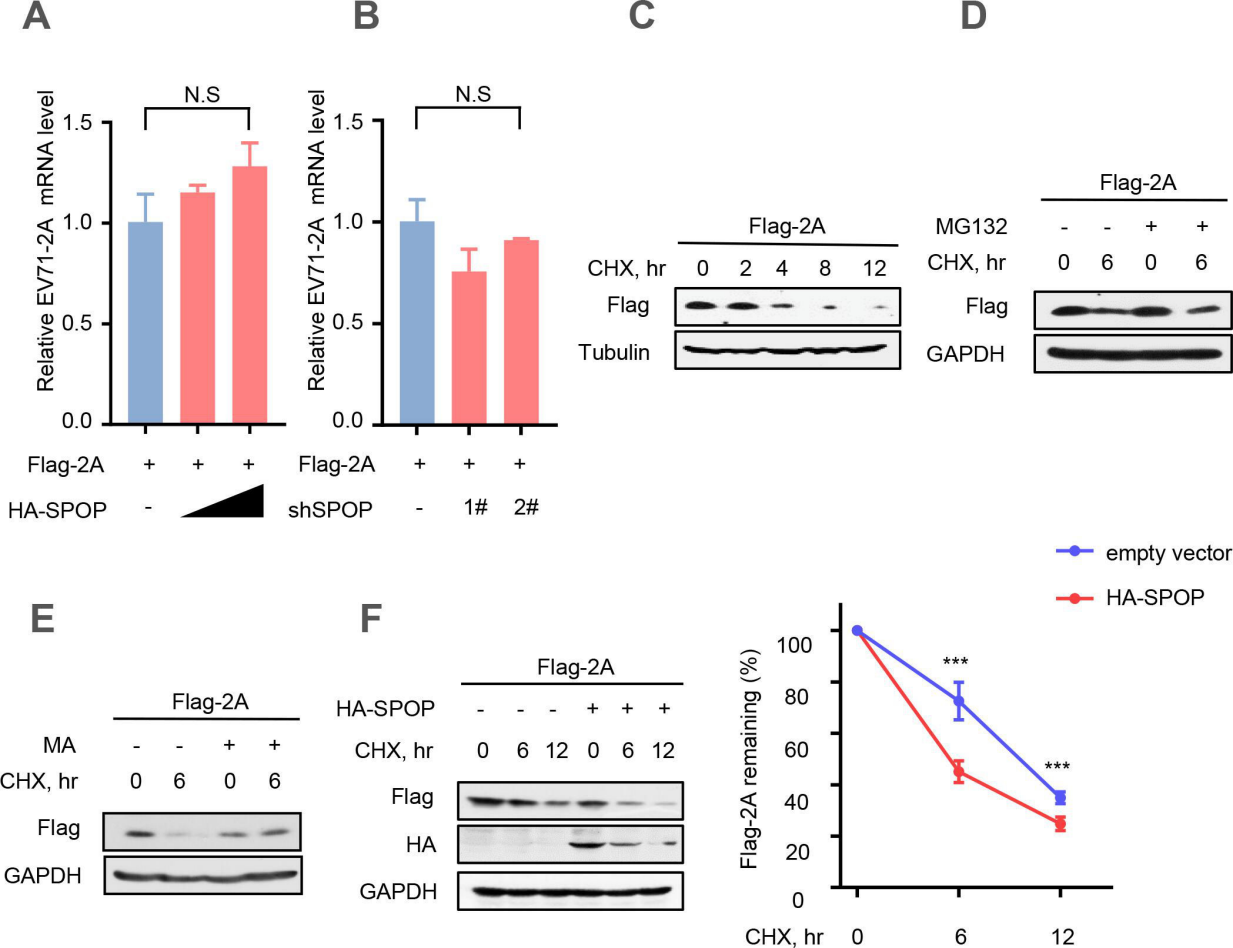
SPOP lowers EV71-2A^pro^ protein stability. (**A**) The relative mRNA level of EV71-2A^pro^ was measured by RT-qPCR in HEK293T cells co-transfected with the vectors to express Flag-2A and empty vectors (−) or HA-SPOP. (**B**) The relative mRNA level of EV71-2A^pro^ was measured by RT‒qPCR in HEK293T cells co-transfected with vectors to express Flag-2A and control shRNAs (−) or shSPOP (1# and 2#). (**C**) HEK293T cells were transfected with a vector to express Flag-2A. Thirty-six hours after transfection, cells were treated with CHX (100 µg/mL) for 0, 2, 4, 8, and 12 hours. The protein levels of Flag-2A were analyzed by immunoblotting. (**D**) HEK293T cells transfected with Flag-2A plasmids were pretreated with CHX (100 µg/mL) for 6 hours and then treated with DMSO or MG132 (10 µM) for 4 hours. The protein levels of Flag-2A were analyzed by immunoblotting. (**E**) HEK293T cells transfected with Flag-2A plasmids were pretreated with CHX (100 µg/mL) for 6 hours and then treated with DMSO or MA (20 mM) for 8 hours. The protein levels of Flag-2A were analyzed by immunoblotting. (**F**) HEK293T cells were transfected with either empty vectors (CON) or HA-SPOP plasmids, together with Flag-2A plasmids. Then, the cells were treated with CHX for 0, 6, and 12 hours. Whole-cell extracts were analyzed by immunoblotting using the indicated antibodies (left). Data were analyzed by GraphPad Prism 7 (right). N.S., not significant (*P* > 0.05) and ***P < 0.001 (two-tailed unpaired Student’s *t*-test). Data are representative of three independent experiments (**C–F**) or shown as the mean and s.d. of three biological replicates (**A and B**).

Based on the aforementioned findings, our subsequent objective was to investigate whether SPOP downregulates the levels of EV71-2A^pro^ by affecting EV71-2A^pro^ transcription. Therefore, HEK293T cells were co-transfected with the vectors expressing Flag-2A or HA-SPOP. The results showed that overexpression of SPOP did not affect the mRNA level of EV71-2A^pro^ (Fig. 3A). Furthermore, knockdown of SPOP using two specific shSPOP sequences did not exert any influence on the mRNA levels of EV71-2A^pro^ (Fig. 3B). Next, we employed cycloheximide (CHX) (46), a known inhibitor of protein biosynthesis, to assess the stability of EV71-2A^pro^ in cells through a CHX pulse chase assay (Fig. 3C). In addition, we utilized the proteasome inhibitor MG132 (47) to investigate whether the SPOP-mediated degradation of EV71-2A^pro^ occurs via the proteasome pathway. The findings revealed that treatment with MG132 did not impede the SPOP-induced degradation of EV71-2A^pro^ (Fig. 3D). Next, we used methyladenine (MA) (48), a commonly used lysosomal inhibitor, to further explore the mechanisms. Our results showed that MA effectively inhibited the degradation of EV71-2A^pro^ induced by SPOP, suggesting that EV71-2A^pro^ may undergo degradation via the lysosome pathway (Fig. 3E). Furthermore, our observations demonstrated that overexpression of SPOP markedly reduced the protein stability of EV71-2A^pro^ (Fig. 3F). Thus, we demonstrated that SPOP promotes the degradation of EV71-2A^pro^ via the lysosomal pathway.

### SPOP promotes K48-linked ubiquitination and lysosome-dependent degradation of EV71-2A^pro^

**Fig 4:**
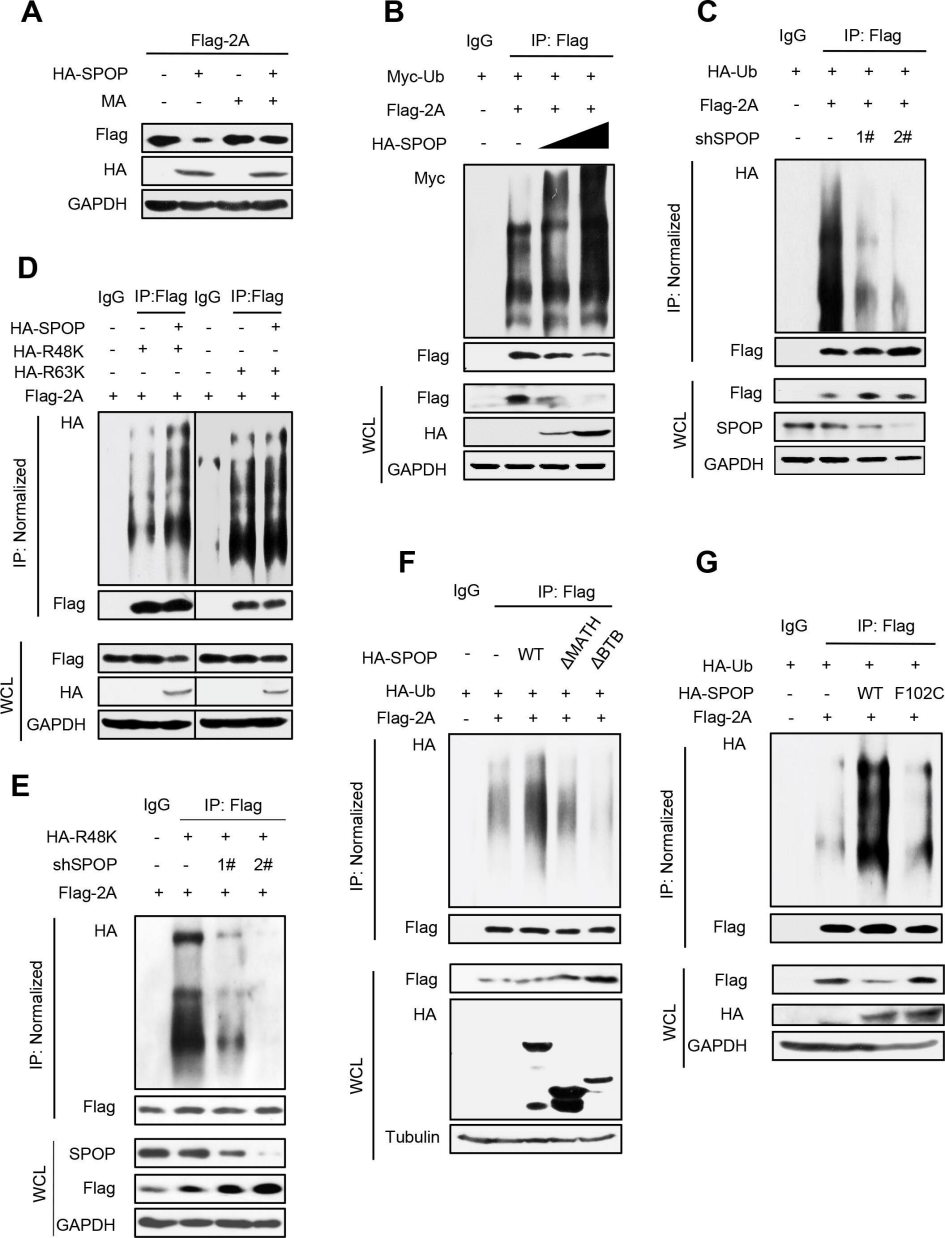
SPOP promotes K48-linked ubiquitination and lysosome-dependent degradation of EV71-2A^pro^. (**A**) HEK293T cells transfected with Flag-2A and/or HA-SPOP plasmids were treated with or without MA (20 mM) for 12 hours before harvest. The protein levels of Flag-2A were analyzed by immunoblotting as indicated. (**B**) HEK293T cells were transfected with the vectors to express Flag-2A together with HA-SPOP and Myc-Ub plasmids as indicated. Flag-2A proteins were immunoprecipitated by Flag beads, and Flag-2A ubiquitination levels were analyzed by immunoblotting. (**C**) HEK293T cells were transfected with control shRNA (−) or shSPOP (1# and 2#) plasmids, together with Flag-2A and HA-Ub plasmids as indicated. Flag-2A proteins were immunoprecipitated by Flag beads, and Flag-2A ubiquitination levels were analyzed by immunoblotting. (**D**) HEK293T cells were transfected with Flag-2A and/or HA-SPOP plasmids, together with HA-Ub-K48 only (R48K) or HA-Ub-K63 only (R63K) plasmids. Flag-2A proteins were immunoprecipitated by Flag beads, and then Flag-2A ubiquitination levels were analyzed by immunoblotting. (**E**) HEK293T cells were transfected with control shRNA (−) or shSPOP (1# and 2#) plasmids, together with Flag-2A and HA-Ub-K48 only (R48K) plasmids as indicated. Flag-2A proteins were immunoprecipitated by Flag beads, and Flag-2A ubiquitination levels were analyzed by immunoblotting. (**F and G**) HEK293T cells were transfected with Flag-2A and either HA-SPOP wild type (WT) or HA-SPOP-ΔMATH mutant or HA-SPOP-ΔBTB mutant or HA-SPOP-F102C mutant plasmids, together with HA-Ub plasmids. Flag-2A proteins were immunoprecipitated by Flag beads, and then Flag-2A ubiquitination levels were analyzed by immunoblotting. Data are representative of three independent experiments with similar results (**A–F**).

Given the observed decrease in EV71-2A^pro^ protein stability caused by SPOP, we further investigated the underlying mechanism. Our analysis showed that the administration of MA effectively prevented the downregulation of EV71-2A^pro^ mediated by SPOP (Fig. 4A). Considering the role of SPOP in regulating the lysosome-dependent degradation of EV71-2A^pro^, we subsequently examined whether SPOP also influences the ubiquitination of EV71-2A^pro^. Our results demonstrated that overexpression of SPOP significantly enhanced the polyubiquitination of the Flag-2A protein (Fig. 4B), whereas knockdown of endogenous SPOP markedly inhibited the polyubiquitination of Flag-2A (Fig. 4C). Extensive research has been conducted on two distinct forms of ubiquitination modifications, namely, lysine 48 (K48)-linked (49) and lysine 63 (K63)-linked ubiquitination (50). These modifications have been predominantly employed in the examination of protein ubiquitination. Subsequent investigations demonstrated that overexpression of SPOP notably enhanced K48-linked polyubiquitination of EV71-2A^pro^ but had no significant impact on K63-linked polyubiquitination of EV71-2A^pro^ (Fig. 4D). Furthermore, we performed an experiment to observe whether knockdown of SPOP regulates the K48-linked polyubiquitination of EV71-2A^pro^. The result showed a substantial decrease in K48-linked polyubiquitination of EV71-2A^pro^ when endogenous SPOP was knocked down (Fig. 4E).

Recent reports have demonstrated the significant involvement of the MATH structural domain in substrate recognition and recruitment, as well as the crucial role of the BTB/POZ structural domain in the function of the E3 ubiquitin ligase. Thus, we hypothesized that the removal of either the MATH or BTB/POZ structural domain in SPOP could impact the ubiquitination of EV71-2A^pro^. The results showed that deletion of either the SPOP-MATH or BTB/POZ domains hindered SPOP’s ability to increase the level of polyubiquitination of the Flag-2A protein (Fig. 4F). In addition, we found that SPOP-F102C is incapable of enhancing Flag-2A ubiquitination (Fig. 4G). Thus, these findings suggested that cellular SPOP, functioning as a ubiquitin E3 ligase, induces K48-linked polyubiquitination of EV71-2A^pro^ and promotes the degradation of EV71-2A^pro^ through the lysosome pathway.

### SPOP regulates 2A^pro^ protein levels during EV71 infection

**Fig 5:**
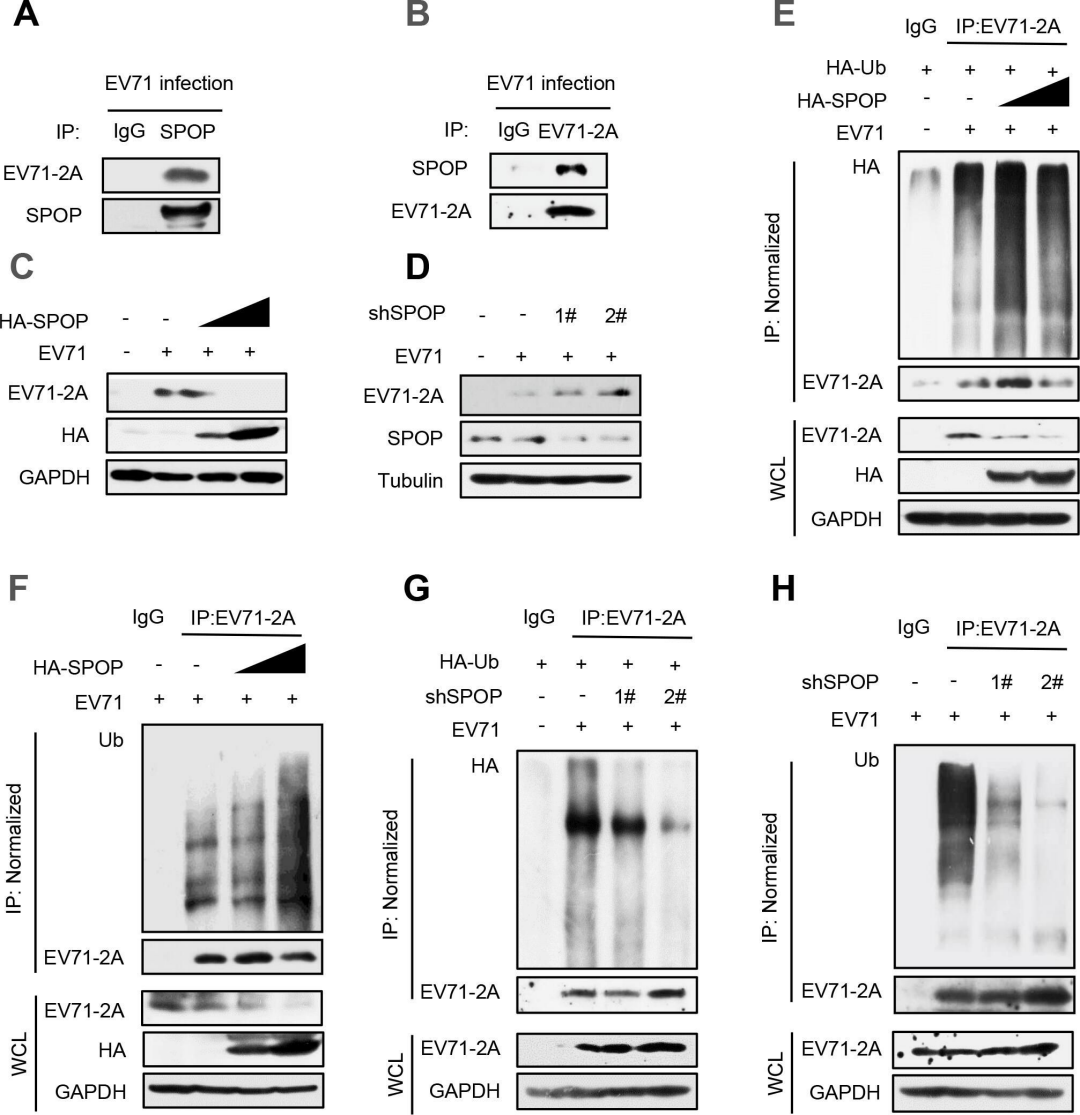
SPOP regulates 2A^pro^ protein levels during EV71 infection. (**A and B**) RD cells were transfected with EV71 for 48 hours to produce EV71-2A^pro^. Immunoprecipitation was performed to analyze the interaction between endogenous SPOP and EV71-produced 2A^pro^ in cells using anti-SPOP antibodies (**A**) or anti-EV71-2A antibodies (**B**). (**C**) RD cells were transfected with EV71 and HA-SPOP plasmids. The protein levels of 2A^pro^ produced by EV71 were analyzed by immunoblotting as indicated. (**D**) RD cells were transfected with EV71 and either control shRNAs (−) or two shSPOP (1# and 2#) plasmids. Whole cell extracts were subjected to immunoblotting as indicated. (**E and F**) RD cells were transfected with HA-SPOP together with HA-Ub plasmids (**E**) or only HA-SPOP (**F**).Immunoprecipitation and immunoblotting were performed to analyze the ubiquitination levels of EV71-2A^pro^ by an anti-HA antibody. (**G and H**) RD cells were transfected with shSPOP together with HA-Ub plasmids (**G**) or only shSPOP (**H**). Immunoprecipitation and immunoblotting were performed to analyze the ubiquitination levels of EV71-2A^pro^ by an anti-Ub antibody. Data are representative of three independent experiments (**A–H**).

We next studied whether SPOP-mediated regulation of EV71-2A^pro^ occurs during EV71 infection. We first found that EV71-encoded 2A^pro^ interacts with cellular SPOP (Fig. 5A and B), which is consistent with the findings presented in Fig. 1. Next, we found that overexpression of SPOP effectively suppressed the expression levels of 2A^pro^ induced by EV71 viruses (Fig. 5C). Conversely, depletion of SPOP in cells resulted in elevated levels of 2A^pro^ produced by EV71 (Fig. 5D). Mechanistically, we demonstrated that overexpression of SPOP significantly increased the cellular ubiquitination levels of 2A^pro^ produced by EV71 viruses (Fig. 5E and G). Consistent with our previous findings, knockdown of SPOP using two specific shRNAs reduced the cellular ubiquitination levels of 2A^pro^ produced by EV71 viruses (Fig. 5F and H). Taken together, these observations demonstrated that cellular SPOP acts as a negative regulator of the level of 2A^pro^ during EV71 virus infection.

### SPOP inhibits EV71 virus infection

**Fig 6:**
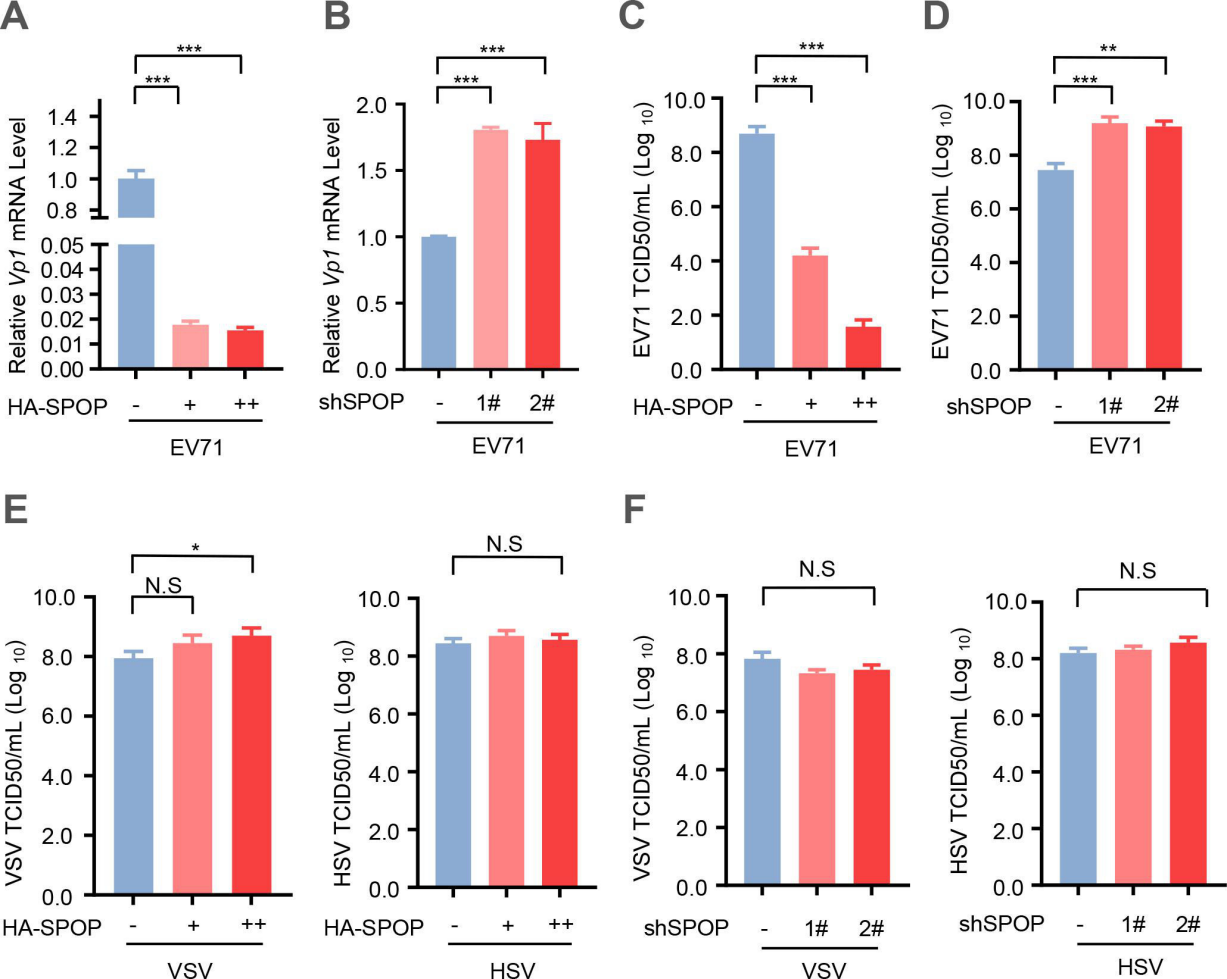
SPOP negatively regulates EV71 virus infection. (**A**) The relative mRNA level of EV71-VP1 was measured by RT-qPCR in RD cells transfected with empty vectors (-) or increased amounts (0.5 and 1.0 µg) of HA-SPOP. (**B**) The relative mRNA level of EV71-VP1 was measured by RT‒qPCR in RD cells transfected with the control shRNAs (−) or shSPOP (1# and 2#). (**C**) RD cells were transfected with empty vectors (−) or increasing amounts (0.5 and 1.0 µg) of HA-SPOP. Then cells were infected with EV71 (MOI = 1.0) for 24 hours. EV71 viral titers in culture supernatants were determined by a TCID-50 assay. (**D**) RD cells were transfected with the control shRNAs (-) or shSPOP (1# and 2#). Then cells were infected with EV71 (MOI = 1.0) for 24 hours. EV71 viral titers in culture supernatants were determined by a TCID-50 assay. (**E**) RD cells were transfected with empty vectors (-) or increasing amounts (0.5 and 1.0 µg) of HA-SPOP. Then cells were infected with VSV or HSV (MOI = 1.0) for 24 hours. VSV and HSV viral titers in culture supernatants were determined by a TCID-50 assay. (**F**) RD cells were transfected with the control shRNAs (−) or shSPOP (1# and 2#). Then cells were infected with VSV or HSV (MOI = 1.0) for 24 hours. VSV and HSV viral titers in culture supernatants were determined by a TCID-50 assay. Data were analyzed by GraphPad Prism 7 (right). N.S., not significant (*P* > 0.05); *P < 0.05; **P < 0.01; ***P < 0.001 (two-tailed unpaired Student’s *t*-test). Data are representative of three independent experiments (**A–F**).

Based on the above findings, we next investigated the potential influence of SPOP on EV71 virus infection. The result showed a significant reduction in EV71 infection upon overexpression of SPOP (Fig. 6A). Conversely, knockdown of endogenous SPOP resulted in an enhanced susceptibility to EV71 infection (Fig. 6B). Additionally, overexpression of SPOP led to a decrease in viral titers in EV71-infected cells (Fig. 6C), whereas depletion of endogenous SPOP resulted in an elevation of viral titers in EV71-infected cells (Fig. 6D). In addition, we observed cellular infection with two additional viruses, namely, the RNA virus vesicular stomatitis virus (VSV) and the DNA virus herpes simplex virus (HSV). The results indicated that neither overexpression nor knockdown of SPOP can affect cellular infection with either VSV or HSV (Fig. 6E and F). Together, these findings demonstrated that SPOP functions as an inhibitor of EV71 virus infection.

## DISCUSSION

**Fig 7:**
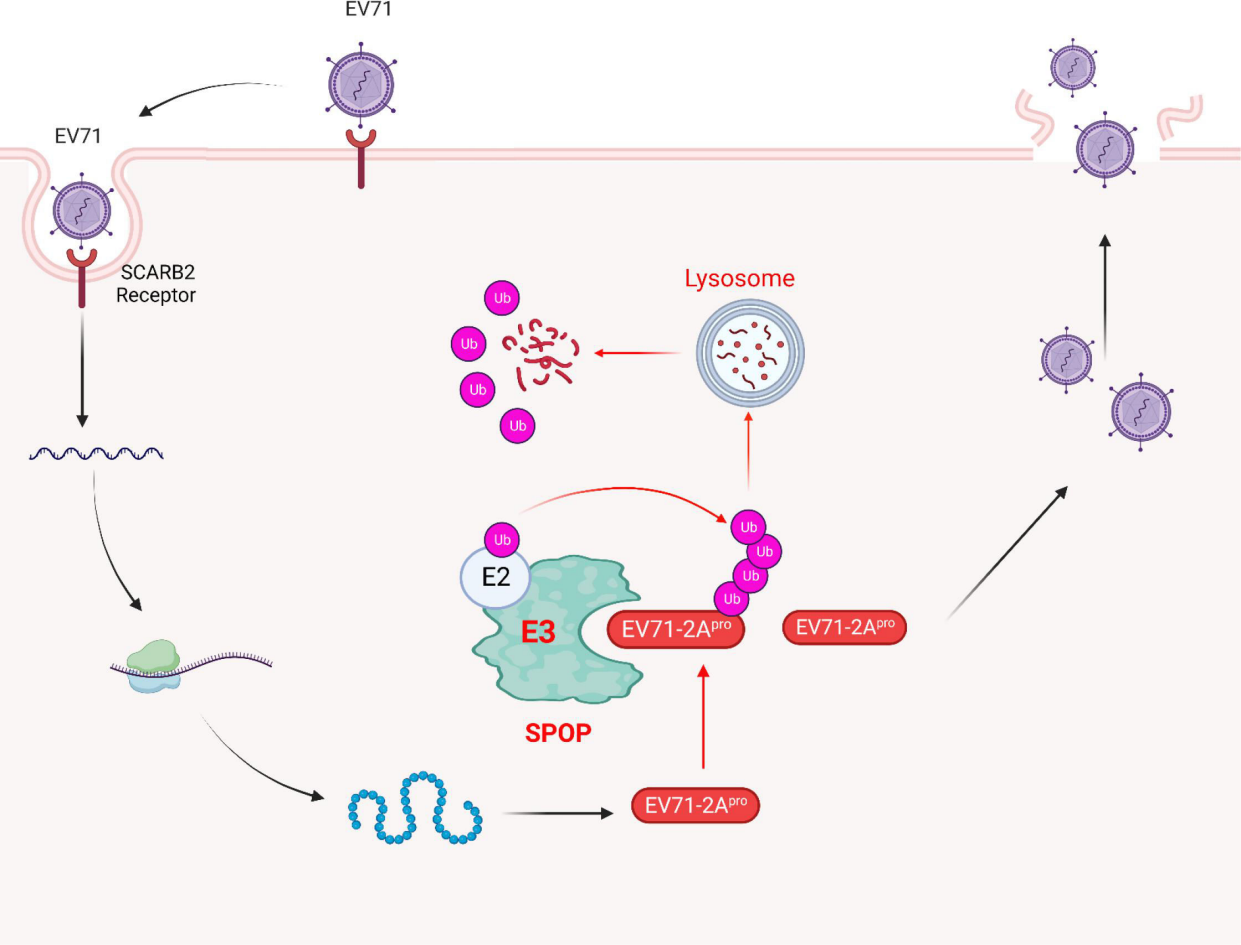
A model for SPOP-mediated inhibition of EV71 infection. EV71 viruses encode 2A^pro^ to aid packaging to produce viral particles. Host ubiquitin E3 ligase SPOP interacts with EV71-2A^pro^ and induces K48-linked polyubiquitination of EV71-2A^pro^, which results in the degradation of EV71-2A^pro^ via the lysosomal pathway. SPOP-mediated degradation of EV71-2A^pro^ ultimately inhibits EV71 infection. Created with BioRender.com.

In prior studies, researchers have presented evidence indicating that the activities of EV71-2A^pro^ are associated with the cleavage of host eIF4G, thereby facilitating EV71 replication, as well as the suppression of various host proteins to counteract immune responses. Nonetheless, the mechanisms by which host antiviral factors impede the functioning of EV71-2A^pro^ remain unclear. This investigation has unveiled the involvement of SPOP in the targeting of EV71-2A^pro^ and subsequent reduction of EV71-2A^pro^ protein levels in cells. Subsequent investigations have elucidated the intricate mechanism by which SPOP facilitates K48-linked ubiquitination and lysosome degradation of EV71-2A^pro^ dependently on its ubiquitin E3 ligase activity. Notably, host SPOP exhibits the ability to diminish the production of EV71-2A^pro^ by EV71 virions. Thus, this study unveiled an important antiviral mechanism by which the host ubiquitin E3 ligase SPOP curtails EV71-2A^pro^ and restricts the virulence of EV71 (Fig. 7).

MAVS and MDA5 are responsible for detecting viral nucleic acids and subsequently initiating the expression of host IFN-I (51, 52). Consequently, MDA5 and MAVS play critical roles in defending against picornavirus infection. IFNAR1, which interacts with IFN-I, triggers antiviral responses induced by IFN-I (53). Therefore, maintaining appropriate levels of IFNAR1 is crucial for regulating viral replication. Recent research has demonstrated that EV71-2A^pro^ can suppress the expression of host MAVS protein, MDA5 protein, and IFNAR1 protein, ultimately facilitating EV71 infection (25, 26). Having demonstrated the involvement of SPOP in the regulation of EV71-2A^pro^ protein levels, we think that the downregulation of EV71-2A^pro^ by SPOP could effectively hinder EV71 infection by enhancing the antiviral activity mediated by MAVS, MDA5, and IFNAR1. Our observations further supported the inhibitory effect of SPOP on EV71 infectivity. It is important to note that other regulatory factors may also contribute to the inhibition of EV71 infectivity mediated by SPOP. Collectively, these findings contributed to a better understanding of the roles played by both SPOP and EV71-2A^pro^ in the regulation of EV71 infectivity.

The involvement of the ubiquitin system in the regulation of the EV71 life cycle has been documented. For instance, ARRDC4 has been shown to decrease EV71 infection by facilitating K63-linked polyubiquitination of MDA5 through TRIM65 (54). USP19 suppresses cellular type I interferon signaling by deubiquitinating TRAF3, thereby promoting EV71 infection (4). Additionally, USP4, functioning as a deubiquitinating enzyme, can target TRAF6 for K48-linked deubiquitination, leading to the positive regulation of RLR-induced NF-κB activation and the inhibition of EV71 replication (55). Recent studies have demonstrated that ubiquitin-specific protease 24 plays a crucial role in facilitating EV71 infection by impeding the K63-linked polyubiquitination of TBK1 (3). In addition, TBK1 also functions as an E3 ubiquitin ligase to regulate the phosphorylation and ubiquitination of multiple picornavirus VP3 proteins (56). Nevertheless, there is currently no literature documenting the direct impact of E3 ubiquitin ligases on the expression of nonstructural proteins encoded by EV71. In this study, we found that SPOP induces ubiquitination and lysosome-dependent degradation of EV71-2A^pro^, leading to the inhibition of EV71 infection. It has been reported that the lysosomal pathway can be affected by enterovirus replication due to the action of viral proteases (57 – 59). Here, this study revealed that EV71-encoded 2A protease can be degraded by the host ubiquitin-lysosomal pathway. Ubiquitin has been widely suggested to have a role in the process of selective autophagy (60, 61), particularly as a signal for the degradation of substrate proteins via the lysosomal pathway through the K48 polyubiquitin chain (62, 63). Our study showed that SPOP induced K48-linked polyubiquitination of EV71-2A^pro^ and that the lysosomal inhibitor 3-methyladenine effectively impeded SPOP-mediated degradation of EV71-2A^pro^, demonstrating the effect of the ubiquitin-lysosome pathway on EV71-2A^pro^ degradation. These findings provided clues for future investigations into the interplay between EV-71-induced alterations in the lysosomal pathway and the degradation of EV71-2A^pro^ mediated by host factors.

The initial identification of SPOP stemmed from its correlation with prevalent mutations observed in prostate and endometrial cancers, especially within the MATH domain, leading to the destruction or decrease of substrate affinity. In our follow-up study, we found that in addition to EV71 viruses, the 2A proteases encoded by Coxsackievirus groups A2, A4, A6, A10, A12, and A16 have VSSTT amino acid residues, suggesting that SPOP may inhibit the level of the 2A proteases encoded by these viruses. This would provide a potential basis for the inhibition of enterovirus infection by SPOP. A recent investigation has shed light on the significant contributions of SPOP to innate immunity. Specifically, SPOP exerts a restraining effect on inflammation by ubiquitination of the innate signal transducer Myd88 (32). Furthermore, SPOP is implicated in the regulation ofinterferon regulatory factor (IRF) activity induced by Toll-like receptor (TLR) (39). These findings collectively indicated that SPOP functions as a suppressor of host immune responses. In this study, it was observed that SPOP has the capability to decrease the levels of EV71-2A^pro^, consequently impeding the progression of EV71 infection. It is worth noting that the SPOP gene often undergoes mutations in cancers, especially within the MATH domain, leading to the destruction or decrease of substrate affinity. SPOP mutations in cells could affect the degradation of EV71-2A^pro^ and therefore could exacerbate EV71 infection.

However, a dearth of dependable animal models exists for the examination of disease manifestations in humans caused by EV71 infection (64, 65). Notably, the induction of EV71 infection in mice under experimental conditions was contingent upon both the age of the mice and the dosage of the infection (66, 67). Following intraperitoneal inoculation, mortality reached 100% in 1-day-old ICR mice, with a subsequent decline observed as the age at inoculation increased (68). The limitations of this animal model lie in the fact that 1-day-old lactating EV71 mice are too young for antiviral testing. Additional models of mouse infection have been developed through the successive infection of mice to generate mouse-adapted strains of EV71 or by creating adult immunodeficient mice (67, 69). Nonetheless, the limited ability of mouse-adapted strains to infect mice beyond 14 days of age and the existence of an inherent predisposition bias render this animal model imperfect (70). However, transgenic mouse models failed to support efficient oral (intragastric) infection, which is a major route for transmission in children. The occurrence of pulmonary edema and the consequent rapid onset of cardiopulmonary failure are distinctive features of mortality induced by EV71 (71). Regrettably, none of the existing mouse models demonstrate pulmonary edema (72). Consequently, the utility of these murine models in investigating the disease mechanism of EV71 is limited (64). Consequently, we anticipate the development of a mouse model that mimics the infection mechanism observed in humans, which would enable the confirmation of SPOP function *in vivo* during EV71 infection using SPOP-deficient mice.

## MATERIALS AND METHODS

### Cell culture and transfection

The HEK293T, RD, and Vero cell lines were acquired from the American Type Culture Center (ATCC). The cells were cultured at a temperature of 37°C with a 5% CO_2_ atmosphere in Dulbecco’s modified Eagle’s minimal essential medium (DMEM) supplemented with 10% fetal bovine serum (FBS), 100 U/mL penicillin, and 100 U/mL streptomycin. All transient transfections were conducted using LongTrans (Ucallm, China) following the guidelines provided by the manufacturer.

### Plasmids and reagents

Plasmids expressing EV71 virus proteins, including Flag-tagged 2A (Flag-2A), Flag-tagged 2B (Flag-2B) and Flag-tagged 2C (Flag-2C), were synthesized by TranSheep (Shanghai, China); the plasmid vector was pIRES2-EGFP, and the promoter was the CMV promoter. HA-SPOP, shSPOP 1#, shSPOP 2#, HA-ubiquitin (HA-Ub), Myc-ubiquitin (Myc-Ub), HA-R48K, and HA-R63K (all lysins on the ubiquitin gene were mutated to arginines except the corresponding lysine) were gifts from Dr. Hui Zheng (Soochow University, China) (38, 73 – 75). The HA-SPOP-F102C and Flag-2A-ΔVSSTT mutants were generated by a Quick-Change Site-Directed Mutagenesis Kit (Stratagene, America). SPOP-ΔMATH (Lys28-Asp166) was made using PCR amplification from the HA-SPOP plasmids. All shRNAs were constructed using the RNAi-Ready p-SIREN-RetroQ-ZsGreen vector. All of the plasmids were confirmed by DNA sequencing. CHX, MA, MG132, and other chemicals were purchased from Sigma (America).

### RNA isolation and RT-qPCR

Total RNA was extracted from cells using TRIzol reagent (Invitrogen). All cDNA was synthesized from 500 ng of total RNA using 5× All-in-One RT Master Mix (Abcam, America). Then, mRNA levels were analyzed by RT-qPCR using 2× SYBR Green qPCR Master Mix (Selleck, cat. no. B21202). The GenBank accession numbers for SPOP and β-actin are AJ000644 and AK025375, respectively. The β-actin primers were designed to target the nucleotide positions 229–252/398–421, and the SPOP primers were designed to target the nucleotide positions 195–217/278–299. The GenBank accession number for EV71-2A is AB550332. The EV71-2A primers were designed to target nucleotide positions 48–67/128–147.

The detailed primer sequences were as follows:

EV71-2A^pro^: 5′-agtggttaaccgccatcttg-3′ and 5′-accttgggcagtggtagatg-3′

SPOP: 5′-gaaatggtgtttgcgagtaaacc-3′ and 5′-gcccgaacttcactctttgga-3′

VP1: 5′-gagtggcagatgtgattga-3′ and 5′-tccagtgtctaagcgatga-3′

β-actin: 5′-accaactgggacgacatggagaaa-3′ and 5′-atagcacagcctggatagcaacg-3′

### Viral infection

The EV71 strain (GenBank accession number: AB550332.1) came from the China Centre for Type Culture Collection. The VSV was a gift from Dr. Chen Wang (Shanghai Institutes for Biological Sciences, Chinese Academy of Science). The erpes simplex virus was a gift from Dr. Chunfu Zheng (Fujian Medical University, China). Briefly, cells were transfected with HA-SPOP or shSPOP plasmid for 36 or 60 hours and EV71-infected cells for 2 hours with DMEM. Then, the DMEM was replaced with a fresh medium containing 10% FBS for 24 hours.

### Cycloheximide pulse chase assay

Flag-2A stability was detected by CHX pulse chase assay (46). Briefly, HEK293T cells were co-transfected with HA-SPOP and Flag-2A plasmids. After transfection for 36 hours, the cells were treated with CHX (100 µg/mL) or DMSO for the indicated times. Subsequently, the cells were collected and detected by Western blotting.

### Immunoprecipitation assay and immunoblotting

After transfection with the indicated plasmid for 48 or 72 hours, cells were collected by using lysis buffer containing 150 mM NaCl, 1% Nonidet P-40, 0.5 mM EDTA, 20 mM Tris-HCl (pH 7.4), and PMSF (50 µg/mL). Next, the appropriate antibodies were added to the cell lysates and centrifuged at 4°C overnight. Then, the cell lysates were added to the Protein G beads, spun at 4°C for 2 to 3 hours and washed several times using a washing buffer. Finally, a 3× loading buffer was added to release the proteins from the beads, and the proteins were analyzed by Western blotting. For immunoprecipitation of Flag/HA-tagged proteins, M2 affinity gel (A2220; Sigma‒Aldrich) and HA magnetic beads (Selleck) were added to cell lysates. Then, the lysates were rotated for 3 hours on a rotor at 4°C. After washing three times with a washing buffer (150 mM NaCl), a 3× loading buffer was added and the samples were heated at 95 ℃ to release the proteins from the beads. Then, the proteins were analyzed by Western blotting.

A total of 1%–2% of the input lysates of whole cells served as a control. The following antibodies were used: SPOP (Abcam, ab192233, 1:1,000), EV71/VP1 (Abcam, ab169442, 1:1,000), HA (Abcam, ab9110, 1:3,000), VSV-G (Abcam, ab1874, 1:1,000), Flag (Sigma, F7425, 1:1,000), GAPDH (Good here Biological Technology, AB-M-M001, 1:5,000), anti-tubulin (Proteintech, 66031–1-Ig, 1:5,000), HRP-conjugated goat anti-rabbit IgG secondary antibodies (Proteintech, SA00001-2, 1:5,000), HRP-conjugated goat anti-mouse IgG secondary antibodies (Proteintech, SA00001-1, 1:5,000), Myc (Abmart, m2002, 1:3,000), anti-mouse IgG (Abmart, A25012, 1:2000), and anti-rabbit IgG (Abmart, A25022, 1:2,000).

The EV71-2A^pro^ antibody was generated by You Ke (Shanghai) through immunization of rabbits with pET28a-GKFGQQSGAIYVGNFRVVNRHLATHNDWANLVWEDSS

RDLLVSSTTAQGCDTIARCNCQTGVYYCNSMRKHYPVSFSKPSLIFVEASEYYPARYQSHLMLAVGHSEPGDCGGILRC peptide.

When protein ubiquitination was analyzed, N-ethylmaleimide (10 mM) was added to the above lysis buffer. The immunoprecipitates were washed twice using normal washing buffer (150 mM NaCl) and subsequently with high-salt (550 mM NaCl) washing buffer three times. Finally, 3× loading buffer was added and the samples are heated at 95℃. Then, the proteins were analyzed by Western blotting.

### Immunofluorescence and confocal microscopy assay

Cells were infected with VSV-GFP for 24 hours. Then, cells were analyzed under an immunofluorescence microscope. The images were magnified at 200×. In the confocal microscopy assay, cells were transfected with the indicated plasmid for 48 hours. After washing with 1× PBS five times, cells were fixed with 4% paraformaldehyde on ice for 1 hour. Then, cells were permeabilized with 0.1% Triton X-100 at room temperature for 1 hour and blocked with 3% BSA at room temperature for 1 hour. Cells were incubated with the appropriate antibodies at 4°C overnight. After washing five times with 1× PBS, the cells were stained with 488 goat anti-mouse IgG (Alexa Fluor, A11001) or 594 goat anti-rabbit IgG (Alexa Fluor, A11012). Cell nuclei were stained with DAPI. Fluorescence images were captured with a Nikon A1 confocal microscope.

### TCID50 assay

Cells were first transfected with different constructs. Then, the cells were infected with the virus for 24 hours. Culture supernatants containing virus were serially diluted with DMEM and then placed on a monolayer of Vero cells in 96-well plates. Monolayer positivity for virus infection was noted by cytopathic effects. The TCID50 was calculated using the Spearman-Karber algorithm (74).

### Statistical analysis

Data analysis was performed using GraphPad Prism 7.0 (GraphPad Software, San Diego, CA) software. Different groups were compared by a two-tailed Student’s *t-*test. All differences were regarded as statistically significant when *P < 0.05, **P < 0.01, and ***P < 0.001.

## Data Availability

All the data generated during the current study are included in the manuscript.
